# The Integration of Mental Health and Psychosocial Support and Disaster Risk Reduction: A Mapping and Review

**DOI:** 10.3390/ijerph17061900

**Published:** 2020-03-14

**Authors:** Brandon Gray, Fahmy Hanna, Lennart Reifels

**Affiliations:** 1 Department of Psychological Science, University of Vermont, Burlington, VT 05401, USA; 2 Department of Mental Health and Substance Use, World Health Organization, 1202 Geneva, Switzerland; hannaf@who.int; 3 Centre for Mental Health, Melbourne School of Population and Global Health, The University of Melbourne, Melbourne VIC 3010, Australia; l.reifels@unimelb.edu.au

**Keywords:** Mental Health and Psychosocial Support, Disaster Mental Health, Disaster Risk Reduction, Disaster Risk Management

## Abstract

The field of disaster and emergency management has shifted in focus towards the goal of Disaster Risk Reduction (DRR). However, the degree to which the Mental Health and Psychosocial Support (MHPSS) field has followed this trend is relatively unknown. Therefore, the objectives of this review were to identify relevant projects, materials, and publications relating to MHPSS and DRR integration and define current domains of action in this integration. A review was conducted using a two-pronged approach for data collection. This approach included 1) a mapping exercise eliciting relevant documentation and project descriptions from MHPSS actors, and 2) a database and internet literature search. The mapping exercise was conducted between January and November 2019, while the literature search was completed in March 2019. The majority of identified materials concerned actions of capacity and systems building; preparedness; policy development, consensus building, and awareness raising; school- and child-focused DRR; inclusive DRR; and resilience promotion. Results also suggested that relatively little consensus exists in terms of formal definitions of and frameworks or guidance for integrating MHPSS and DRR. Moreover, domains of action varied in terms of current implementation practices and empirical evidence. Materials and projects are reviewed and discussed in terms of implications for advancing the integration of DRR and MHPSS and expanding MHPSS approaches to include building better before emergencies.

## 1. Introduction

Recently, the field of emergency and disaster management has shifted in focus towards the goal of Disaster Risk Reduction (DRR) through Disaster Risk Management (DRM). The 2015–2030 Sendai Framework for Disaster Risk Reduction formally highlighted this continued paradigmatic shift in focus and represents a globally agreed upon model for engaging in DRR practices [1]. This framework, which incorporated 35 explicit mentions of health, emphasizes risk reduction and increased resilience through person-centered and an all-hazard, all-state, and society approach. However, the degree to which the Mental Health and Psychosocial Support (MHPSS) field has followed this proactive trend is relatively unknown. Traditionally, MHPSS services have been primarily focused on the response and recovery phases of emergencies [2]. In the past decade, the World Health Organization (WHO) has taken a leading role in the development, study, and dissemination of best-practice materials and provision of technical field support for MHPSS operations. Experience from these activities has suggested a clear need for guidance on developing MHPSS programming from a DRR perspective. Moreover, research has indicated that academic actors may be more familiar with concepts of the Sendai Framework than practitioners [3] and that significant knowledge gaps exist in approaches to managing MHPSS programming across emergency phases [4]. Therefore, the WHO initiated a three-year project to develop and test practical guidance and curriculum for psychosocial support preparedness in selected countries. This guidance was also commissioned by the Inter-Agency Standing Committee Reference Group on MHPSS in Emergency Settings (IASC RG, which the WHO is currently co-chairing) in order to design a replicable model for preparing psychosocial support in advance of disasters and emergencies globally. In order to further understand the state of integration between the DRR and MHPSS fields and inform this guidance and curriculum, a mapping exercise and literature review were first undertaken. The current review is a product of these efforts. 

### Linking MHPSS and DRR

DRR activities are not narrowly defined and may include “any physical construction to reduce or avoid possible impacts of hazards, or application of engineering techniques to achieve hazard resistance and resilience in structures or systems.” These are also known as ‘structural’ activities in DRR terminology. DRR activities may also include “any measure not involving physical construction that uses knowledge, practice or agreement to reduce risks and impacts, in particular through policies and laws, public awareness raising, training and education.” These are also known as ‘non-structural’ activities in DRR terminology [5].

Strong arguments have been made for linking MHPSS and DRR activities in both ‘structural’ and ‘non-structural’ domains [6] and for shifting paradigms in the field of MHPSS towards “Upstream” approaches targeting preparedness and prevention [2]. Furthermore mental health and well-being are explicitly addressed in the Sendai Framework Priority for Action Area Four [7,8] and further examination of Sendai priorities and indicators suggests that MHPSS services may be relevant to all four Sendai priorities and for indicators A-2, A-3, B-2, D-2, and D-7 [9]. Nonetheless, the integration of MHPSS has seemingly not been comprehensively outlined in DRR theory, policy, or strategy. Furthermore, challenges exist in building consensus agreement on effective strategies for Disaster Mental Health risk reduction [9] and for studying long-term impacts [10]. 

Thus, the current review was intended to provide a narrative exploration of current practices, projects, materials and conceptualizations integrating MHPSS components and DRR approaches or principles primarily in the preparedness and prevention phases. This focus was chosen due to the relative abundance of existing literature discussing MHPSS response and recovery approaches [11,12,13,14,15] and in order to address the 2015–2030 Sendai Framework for Disaster Risk Reduction’s challenge to expand more “upstream” approaches. More specifically, the objectives of this review were to (1) identify existing published and unpublished literature; completed, in progress, or planned projects and operations; and available guidance or training materials related to MHPSS and DRR integration, (2) determine the current domains of action where MHPSS and DRR have been integrated, and (3) discuss potential avenues for further integrating these two fields. 

## 2. Materials and Methods 

Because the overall extent to which actors in the field have developed DRR programming with designated MHPSS components (and vice versa) was relatively unknown, a narrative review and discussion of the literature was undertaken. This review approach allows for a survey of the current state of knowledge of a particular topic and is suitable for understanding topics with limited or unknown parameters [16]. This review was conducted using a two-pronged approach that drew on two main data sources.

First, a mapping exercise was initiated by the WHO and lead by the first author in January 2019 using a newly constructed mapping tool intended to identify *who* was doing *what*, *when*, and *where* in the area of MHPSS and DRR following the ‘4ws’ approach [17] and adapted to the Sendai Framework priorities for action [1]. As part of this initiative, all respondents were asked to provide both published and unpublished “grey” literature (which included any internal formal or informal frameworks, guidelines, or other relevant materials) as well as project descriptions and operational summaries of any completed, in-progress, or planned initiatives that concerned MHPSS prevention and preparedness and/or related DRR/DRM approaches. In initiating the mapping exercise, all member organizations of the IASC MHPSS RG (56 members) were contacted. Established in 2007, the IASC MHPSS RG fosters a unique collaboration between non-governmental organizations (NGOs), United Nations and international agencies and academics in an effort to promote best practices in MHPSS. The IASC MHPSS RG is co-chaired by the WHO and IFRC and includes a global representation of members working at local, regional, national, and international levels. The group has the mandate of developing and disseminating interagency guidance on MHPSS, mainstreaming MHPSS in humanitarian settings and supporting country level MHPSS Technical Working Groups. Country-level MHPSS Technical Working Groups, co-led by different organizations (e.g., national NGOs, international NGOs and/ or UN agencies) in cooperation with relevant Government Line Ministries exist currently in 21 humanitarians, migrants and refugees settings, and all are supported by the Reference Group through technical guidance, country level missions, global meetings for group co-chairs and regular teleconferences [18]. Additionally, multiple academic actors and institutions, and other relevant non-governmental organizations (NGO) and service providers were contacted. Referral sampling was also employed in order to expand the reach of the initiative. The mapping initiative and tool for data collection was also posted on MHPSS.net and distributed through social media platforms and email list services in order to generate awareness. 

Second, a systematic database and internet literature search was conducted in order to further identify relevant records. This approach involved combining Medical Subject Headings (MeSHs) with terminology that relates to MHPSS, DRR, and preparedness and prevention. Keywords included Disaster Risk Reduction, Hazard Reduction, Disaster Risk Management, Mental Health and Psychosocial Support, Psychosocial Well-Being, Psychological Health and Psychological Resilience. Databases included MEDLINE (R), PsycINFO, PsycARTICLES Full Text, PsycEXTRA, Cochrane, PubMed and CINAHL. Additionally, internet searches of Google and Google Scholar were conducted. Table 1 summarizes the search strategy and search terms.

Inclusion was limited to published academic and unpublished “grey” literature, documents, materials, or projects relating to MHPSS that included a focus on DRR, emergency or disaster preparedness or emergency or Disaster Risk Management. The type of publication or document was not limited. Inclusion criteria were as follows:

- Focus: Materials or projects that involve Disaster Risk Management or reduction perspectives or actions as well as mental health and/or psychosocial support components or actions.
- Scope: Unlimited; included guidance documents, guidelines, recommendations, consensus standards, research articles, internal organization frameworks, case studies, policy documents, etc.

Because the focus of this review was to identify resources related to MHPSS and DRR integration focused in the preparedness and prevention phases, materials or resources that were solely focused on emergency response or recovery phases or limited to post-disaster management were excluded. Also, materials without full-text availability in English were excluded. Additionally, community resilience was only outlined in this review. This decision was made because community resilience-focused DRR literature varied widely in terms of both definitions of resilience and in explicit incorporation MHPSS considerations. Because developing a single definition of community resilience was beyond the scope of this review, a comprehensive discussion of resilience-focused DRR resources was not feasible. Therefore, the majority of resources reviewed were focused on MHPSS strategies at the individual level. 

**Figure 1:**
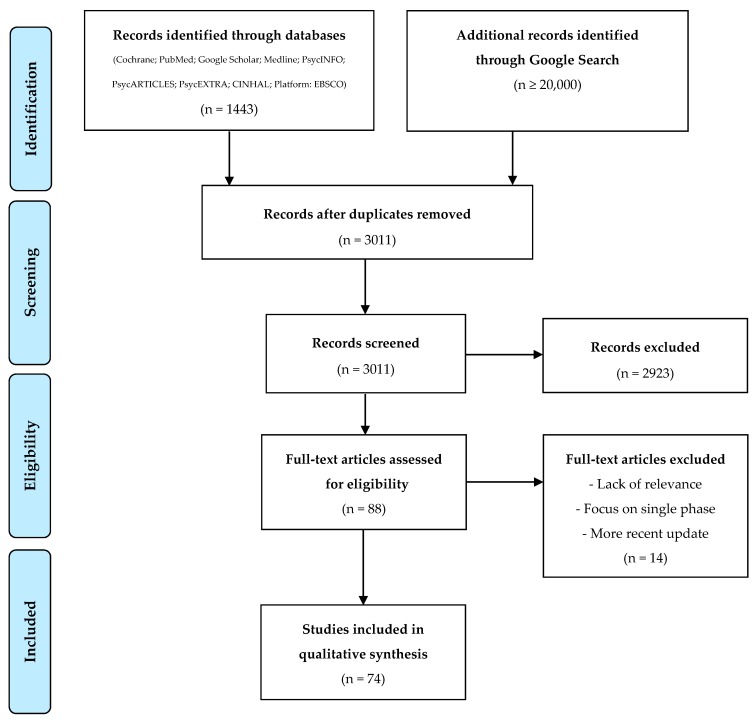
Flowchart for selected resources.

The database search was conducted on March 25th, 2019. Titles of publications were screened, and related abstracts or descriptions reviewed in order to identify resources for full-text review and data extraction (see Figure 1). All full-text reviews were completed using English language versions of resources. Following the mapping exercise and literature review, records were combined for the purpose of narrative synthesis and discussion. 

## 3. Results

### 3.1. Mapping Exercise 

In total, twelve organizations provided completed responses during the mapping exercise. These included seven international non-governmental organizations (INGOs), three United Nations Agencies, one academic institute, and one global platform. Many of the participating organizations reported engaging in international humanitarian operations across many countries and regions. Thus, many organizations relied on responses from regional and local project leaders to provide responses regarding relevant materials and projects. Therefore, these organizations were only able to report on materials and projects identified by these leaders. As a result, relevant materials or projects may exist that have not been included in mapping exercise responses. 

In total, the mapping exercise produced 35 unique records relating to MHPSS and DRR integration. Responses included fourteen project descriptions of completed, current or planned initiatives, five tools or manuals, five conference proceedings or workshop descriptions, and eleven academic publications or conference presentations/symposia.

### 3.2. Database and Internet Search

As shown in Figure 1, over 1446 records were identified through database searches. Additionally, 1568 records were identified using Google searches following removal of duplicate records. From these records, 88 appeared relevant to the purpose of this review during examination of titles, abstracts, or descriptions and were selected for full-text review. During full-text review, 14 records were excluded due to lack of relevance to the topic, a focus primarily on a single phase of disaster response, no explicit reference to DRR related concepts, or the identification of a more recent update of the same publication or material. See Figure 1 for a diagram displaying the search process. 

### 3.3. Current Domains of Integration

Across identified records, a number of themes emerged for classifying the current domains of action where MHPSS and DRR have been integrated. Multiple resources and activities were relevant to many themes. For instance, the Operationalising Psychosocial Support in Crisis Project Comprehensive Guideline on MHPSS in Disaster settings manual was developed for the purpose of aligning MHPSS disaster response operations across European countries [19]. Although explicit to supporting response programming, these guidelines provide a clear and comprehensive insight into practices in planning for all phases of emergencies and across many of the themes identified in this review. Such is the nature of many of the resources discussed herein. However, for the purpose of this review, resources were discussed in terms of the theme(s) that appeared most relevant during full-text review. Further exploration of each resource is recommended for a full accounting of specific areas of relevance. 

#### 3.3.1. Capacity and Systems Building 

Capacity and systems building was a common theme relevant to the integration of MHPSS and DRR across many identified records. Both development of mental health systems and capacity for implementation of specific interventions and approaches were highlighted. Each of these areas and related materials are outlined in the following section. 

Research examining the importance of capacity building for MHPSS service providers was identified in one record. A recent scoping review discussed the role of local faith communities (LFCs) and their contribution to psychosocial well-being, resilience, and DRR as part of the Joint Learning Initiative on Faith and Local Communities [20]. The review of over 300 academic publications, policy documents, and local interviews identified the benefit of including LFCs in DRR activities as well as the importance of increasing their capacity for MHPSS given their unique access to communities.

Manuals were also identified and included guidance for relevant actors in a number of key areas relevant to MHPSS and DRR integration. Among these were examples such as the Pan American Health Organization (PAHO)/WHO’s *Mental Health and Psychosocial Support in Disaster Situations in the Caribbean: Core Knowledge for Emergency Preparedness and Response* publication [21]. These manuals often included discussions of building local capacity through relevant trainings and empowering local communities and actors. Principles and practices of common interventions, such as those of Psychological First Aid (PFA) in line with the *Psychological First Aid: Guide for Field Workers* [22], and the value of preparedness to implement these interventions prior to emergencies were also often discussed. 

Specific projects, such as those identified in Western Africa, also demonstrated the importance of capacity and system building generally. For instance, prior to the 2014–2015 Ebola epidemic, CBM International and other coalition partners had begun working to establish partnerships with the Sierra Leone Ministry of Health and Sanitation [23]. These partnerships were crucial to successfully promoting investment in a sustainable mental health system among various ministries of the Sierra Leone government and led to increased devotion of resources and consideration of MHPSS throughout the country prior to the outbreaks. Following these outbreaks, a global consortium known as READY (2018) was also formed, headed by Save the Children [24]. The purpose of this project has been to build on current technical guidance in various areas to develop a fully integrated disease response framework to serve as a foundation for operational guidance tools, training curriculum and comprehensive learning strategies moving forward. MHPSS service capacity building represents an important component of this project and further demonstrates the integration of a risk reduction perspective. 

Other projects identified focused on the value of increasing capacity for specific approaches or interventions. Capacity for implementing Psychological First Aid (PFA) was common among these projects. For example, the Asian Disaster Preparedness Center (ADPC) launched a training initiative piloted with Building Resources Across Communities (BRAC) Bangladesh that aimed to enhance local capacity of health personnel in assessing and managing psychosocial needs and implementing (PFA) post-disaster [25]. Similarly, in Europe, the Psychological First Aid and Psychosocial Support in Complex Emergencies (PFA-CE), which represents a massive initiative to streamline PFA training and build capacity, completed a desk review documentation of best practices, tools, and recommendations for PFA [26,27] and aims to develop a training of trainers’ package for increasing PFA provision capacity [27]. Likewise, in Bangladesh, the Dhaka Earthquake and Emergency Preparedness (DEEP) project has focused on several areas of disaster preparedness, including basic psychosocial support and PFA as well as stigma reduction in Dhaka City [28,29]. Finally, in the Caribbean, PAHO/WHO has worked to support MHPSS actors through capacity building trainings in interventions such as the Mental Health GAP program’s (mhGAP) Humanitarian Interventions Guide (mhGAP-HIG) [30], in PFA [31], and in country capacity for needs assessment and action planning. 

#### 3.3.2. Preparedness

Preparedness was also an integral aspect of many materials and projects integrating MHPSS and DRR. This is important given the evidence suggesting that preparedness increases coping ability and decreases risk of distress during and after emergencies [32]. However, MHPSS preparedness and prevention efforts are not always prioritized in health care approaches, particularly in countries that experience frequent emergencies [33]. Thus, the following records focused on demonstrating the value of MHPSS preparedness and risk reduction. 

To begin, conferences promoting preparedness were identified in both mapping and literature review records. By way of example, an intercountry conference was held in 2006 discussing the mental health and psychosocial aspects of disaster preparedness with the objective of assisting countries in developing their own preparedness plans [34]. The conference included discussions of MHPSS-related efforts following disasters, country presentations on lessons learnt from the 2004 Southeast Asian tsunami, and discussion of current and planned MHPSS-related disaster preparedness plans. From these proceedings, essential components of such plans and recommendations were outlined and several national-level plans were developed, including those in Bangladesh, Bhutan, Myanmar, and Thailand [33,34,35,36,37,38].

Guidance manuals focused on preparedness with MHPSS components in various settings were also identified. Examples include PAHO/WHO’s previously discussed guidance manual, which also includes comprehensive discussions of preparing an action plan for MHPSS in the event of an emergency [21] and Americares *Disaster Preparedness Planning Guide for Free and Charitable Clinics* [39], which discusses preparedness for natural disasters but in the context of free or charitable health clinics. These manuals outline practical steps for including MHPSS considerations in disaster and emergency preparedness. 

Projects implementing preparedness actions were also noted among records [40,41,42,43,44,45]. These projects included elements such as preparedness planning at various levels (i.e., individual/family, community, national levels) through multi-hazard approaches, establishment of responder rosters and simulation exercises, and training in effective self-help and coping strategies prior to emergencies. Research has indicated that such approaches can be effective in increasing both social cohesion and perceived preparedness and that this increase may account for decreased depression, anxiety, traumatic stress-related symptoms, and functional impairment thereafter [42,43,44].

#### 3.3.3. Policy Development, Consensus Building, and Awareness Raising

The theme of policy development and consensus building also emerged across multiple records captured. Policy placement is key for the mainstreaming of MHPSS and DRR. Likewise, such integration requires consensus on the part of relevant stakeholders in order for widespread implementation to take place. The following section outlines records identified as relevant to both development of these policies and the building of consensus or raising of awareness.

Researchers have attempted to study the consensus and disagreement in the integration of MHPSS and DRR. Reifels conducted several in-depth interviews with experts in the two fields and identified multiple challenges for the integration of MHPSS into broader DRR approaches. These challenges included relative isolation of MHPSS and DRR fields in terms of mutual knowledge, highly specialized terminology, limited health domain specific guidance provided by broad global consensus frameworks (e.g., the Sendai Framework), lack of strong evidence for many MHPSS activities, limited resources and sustainability, and under-recognition of MHPSS stakeholder abilities in DRR planning. However, Reifels also identified several areas of consensus as well as opportunities and methods for integrating MHPSS into DRR projects. Opportunities cited included a shared focus on resilience, health promotion, and community-based and self-help strategies, as well as existing linkages between health and emergency literacy frameworks. Useful strategies for promoting DRR and MHPSS integration included joint prevention planning at multiple levels of policy, providing guidance on funding and policy placement, continuing to build an evidence-base in order to promote policy inclusion, and increased marketing and outreach of MHPSS provisions in emergencies [2,9]. 

Several efforts have already been made or are underway that aim to promote the integration of MHPSS with DRR policies and initiatives and build on these areas of consensus [2,9,46,47,48,49,50,51,52]. For instance, in response the 2014 Ebola crises, experts developed a set of policy recommendations for integrating mental health and psychosocial aspects into policy and planning [53]. Likewise, Valle and CBM International produced a policy brief highlighting the role of mental health in DRR and also in relation to these outbreaks and arguing the cost-effective and efficient nature of strong community-based mental health systems prior to such outbreaks [23]. These efforts represent pivotal steps in ensuring MHPSS is represented in relevant DRR policy.

Additionally, projects were identified that demonstrate efforts to build consensus and raise awareness for MHPSS issues among both community members and policy makers. For example, in an effort to reduce stigma and promote community resilience in the Caribbean, PAHO/WHO and the CDB also implemented an awareness-raising and communication campaign as part of their previously discussed initiative. Known as the *Stronger Together* campaign, the project focused on providing information on effective coping and reducing stigma towards mental health conditions and psychosocial distress. The campaign embedded the “One Love” mentality of Caribbean island cultures and encouraged community members to support one another through radio jingles, video testimonials, public service announcements, illustrated booklets, social media postings, and governmental briefings [54]. 

#### 3.3.4. School-Based and Child-Focused DRR

Several resources, publications, and projects also focused specifically on supporting the needs of children through DRR-focused activities, including the development of a Child-Friendly Sendai Framework for DRR [55]. Many of these efforts aim to reduce the risk posed to children’s mental health during disaster situations while also building resilience to adversity. The following section outlines identified records focused on programmes integrating MHPSS and DDR for children and in schools. 

Several training programmes and manuals have been developed that focus specifically on DRR with children and in school settings and include MHPSS components [56,57,58,59,60,61,62,63,64]. These trainings contain didactic components identifying the importance of preparedness and inclusion of children, sections on implementation of certain key activities, such as child-friendly spaces or interventions targeting psychosocial risk factors (e.g., Child-focused PFA [59]), and recommendations for effective programming. While these trainings vary in target audience (e.g., child protection actors, teachers and educational staff, counselors, programme managers), each includes discussion of psychosocial components within a broader DRR context. Though limited, research supports the efficacy of preparedness training for teachers and in school settings for increasing children’s knowledge and preparedness [65]. 

Similar to these training initiatives, guidelines on specific components of MHPSS and DRR programming in school settings and with children were also identified. Guidelines focused on policy priorities and disaster preparedness as well as conceptual frameworks with ready-to-use planning, development, and monitoring and evaluation tools [66,67]. A technical guidance framework made specifically for integrating DRR with psychosocial components into schools was also identified [68]. Several projects specifically focusing on school-based DRR with MHPSS components were also recorded [68,69]. 

#### 3.3.5. Disability and Inclusive DRR 

Disability and inclusive DRR (IDRR) was yet another theme among materials identified. IDRR is supported in the Sendai Framework with the aim to build the resilience of all people by establishing inclusive practices. Several guidelines and frameworks for IDRR exist [70,71,72,73,74,75,76] and provide crucial guidance given the increased vulnerability of individuals with physical and psychosocial disabilities in disaster settings [77]. The practices and concepts of IDRR are themselves directly aligned with MHPSS principles and practices and also include components specifically targeting mental health and psychosocial aspects of disability and other factors of marginalization. As demonstrated in the newly released IASC Guidelines on Inclusion of Persons with Disabilities in Humanitarian Action [78], it is important to note that MHPSS represents a crosscutting theme when addressing the needs of people with disabilities in an inclusive manner. The following section discusses projects and relevant resources discussing MHPSS components of IDRR. 

Research examining the particular challenges faced by people with intellectual disabilities and those with mental health issues was discussed in one identified record. Specifically, the Norwegian Association of the Disabled (NAD) conducted a rapid literature review of implications for ensuring equal access and inclusion in DRR actions in the Occupied Palestinian Territories and the refugee settings in Lebanon [79]. Broadly, the review discussed both the context in regard to individuals with intellectual disabilities and mental health issues and the protection, prevention and inclusion factors to consider in programming DRR for humanitarian emergencies. The document also outlined best practices, lessons learned, and evidence from DRR planning and humanitarian efforts in the region. 

Training packages and related policy materials discussing the mental health and psychosocial aspects of IDRR were also identified. These resources aimed to discuss psychosocial concerns and mental health needs placed in context of disability, including as an underlying cause of disability, and focused broadly on building capacity for including disability considerations in disaster planning and response implementation. Examples include Handicap International (HI) Nepal’s 2009 publication *Mainstreaming Disability into Disaster Risk Reduction: A Training Manual* [80] and HI’s policy document on inclusive-DRR focused on individuals with disabilities as well as other vulnerable and often overlooked groups [73].

Rigorous examples of implementing the principles of IDRR, such as the European Network for Psychosocial Crisis Management—Assisting Disabled in Case of Disaster (EUNAD, 2013–2014) and European Network for Psychosocial Crisis Management—Assisting Disabled in Case of Disaster—Implementation (EUNAD IP, 2016–2017) projects were also identified [81]. These projects included emphasis on the importance of including mental health and well-being in DRR approaches and ensuring these practices are inclusive of mental and physical disabilities. 

Inclusive DRR materials with MHPSS components have also focused more broadly on the inclusion of other groups who may be vulnerable in disasters. This is reflected in the development of a Gender and DRR Training Package [82], the European and Mediterranean Major Hazards Agreement (EUR-OPA) inclusive guidance for considering the needs of individuals with disabilities [83], guidance on migrants in DRR planning [84] and Save the Children’s inclusive DRR materials and guidance documents focused on inclusive practices for children [85,86,87,88]. 

#### 3.3.6. Resilience Promotion

Resilience promotion was also a theme commonly targeted and discussed in both MHPSS- and DRR-related projects and materials. However, the term has often been used to describe a number of characteristics at multiple levels (e.g., individual, community, or social resilience). Resilience has also been used to describe physical resilience of structures and psychological resilience in the face of adversity. Furthermore, definitions, indicators of, and approaches to building resilience appear to vary considerably. Therefore, concepts of resilience promotion, and particularly community resilience promotion, were not comprehensively reviewed herein. 

Devaney’s “Understanding Resilience” report provided a discussion of the varied definitions of resilience as well as recommendations for building resilience at multiple levels in disaster programming [89]. Recommendations encouraged a focus on building psychological capability (e.g., hope, optimism, self-efficacy); increasing human capital through practical programmes increasing knowledge, including psychological and emotional preparedness; increasing physical resources such as first aid kits and emergency grab bags; and promoting interconnectedness through bonds, social capital, and a sense of community. 

The International Federation of Red Cross and Red Crescent Societies’ Community-Based Disaster Risk Reduction Study of the characteristics of community resilience affirmed many of these recommendations [90]. Characteristics of resilient communities identified included knowledge of risks and ability to build on past experiences, organization, connectedness, in-place infrastructure and services, economic opportunities, and natural assets. Many DRR programmes or materials are particularly focused on building community level resilience [91,92,93,94,95,96,97,98,99,100]. However, there appears to be no agreed upon model or framework for doing so across these discussions. Further alignment may be necessary to clearly define best practices for promoting resilience at various levels. 

## 4. Discussion 

This narrative review focused on identifying current domains of action integrating MHPSS and DRR through reviewing relevant projects, resources, and guidance materials. Major themes identified through literature searches and mapping included capacity and systems building; preparedness; policy development, consensus building and awareness raising; school- and child-focused initiatives; inclusive DRR; and resilience promotion. While many resources were relevant to many or all of these themes, a lack of consensus or overarching explicit framework was also apparent. Throughout each of the materials or projects reviewed, no definition or consensus-based model for discussing the mental health and psychosocial components of DRR was identified. While many resources discussed the two constructs in isolation, few addressed them together or in an explicit fashion. Likewise, few guidelines or manuals that demonstrate steps to operationalize broad recommendations, such as the Sendai Framework, were identified. Therefore, there was also limited consensus or understanding of what activities constituted the integration of MHPSS and DRR. Moreover, there was no clear consensus on MHPSS placement in DRR policy or guidance on best practices for working with various stakeholders to integrate MHPSS with existing DRR programming. As a result, it is likely that evidence-based or consensus-driven definitions and guidelines for integrating MHPSS into DRR programming will be necessary for widespread implementation to occur. 

Many materials also provided broadly-based guidance, were sometimes primarily focused on European or high-income countries, or discussed only specific types of hazards, such as natural hazards. This focus on natural events is consistent with much of the DRR field generally and is reflected in a recent report detailing the lack of DRR guidance and initiatives in conflict contexts [101]. However, many of today’s protracted emergencies and crises are the result of human-caused emergencies, such as conflict or political violence, and occur in low- or middle-income countries, where hazardous events are more common. While many of these resources and guidelines may translate to some extent to other settings, future efforts aimed at specifically building preparedness for and reducing the mental health and psychosocial risks apparent in conflict settings and in contexts where resources are limited are necessary. 

Finally, also apparent from this review was the relative lack of evidence for programmes or recommendations. While many guidelines were clearly based on expert consensus and included evidence-informed or evidence-based components, it appeared that many of the practices identified for integrating MHPSS with DRR suffered from the same lack of empirical evidence [102,103] or slowed uptake of more solidly supported interventions [104,105] faced by MHPSS responses generally. Thus, it is crucial that DRR efforts with MHPSS components be developed with an emphasis on building a solid evidence base in order to support policy inclusion and broader recognition within the DRR field and among other relevant stakeholders. 

Items reviewed also have implications for operationalizing DRR and MHPSS integration at various levels of action. For instance, further efforts are necessary at the global level to mainstream MHPSS as a valued consideration within DRR approaches and likewise to expand awareness of the value of approaching MHPSS action from a DRR perspective. Furthermore, guidance is clearly necessary to direct this integration. Likewise, at the national level, results have indicated at best infrequent and impartial consideration of MHPSS stakeholders or considerations in efforts to reduce disaster risks. While case examples of collaborations between MHPSS and governmental actors exist and were documented, these cases were limited relative to the international acceptance of DRR approaches generally. Results also demonstrated the need for increased attention at the national level to MHPSS in risk reduction and management through increased funding allocation and formal mainstreaming. Findings also clarified the need for further developed curricula and demonstration of tangible approaches to integrating MHPSS and DRR at the local level. Many materials reviewed discussed broad-based principles but were limited in relevance to practical or contextualized implementation. Results, therefore, convey the value of detailed protocols and case descriptions for projects that do address MHPSS and DRR integration so that other actors may benefit from the successes and lessons learned therein. Finally, this review demonstrates clear implications for agencies involved in MHPSS or DRR programming. Given the lack of formal consideration for integrating these two fields, a crucial step of action for any agency may be to identify MHPSS or DRR focal points and encourage collaboration between these two through joint planning, activities, and monitoring.

### Limitations

Despite noteworthy findings, the current review and its results should be taken in light of a number of limitations. First, this review was conducted using a narrative approach and was limited in the number of databases included for conducting the search. Therefore, this review cannot be assumed to be entirely comprehensive. Second, many agencies who may be active in linking MHPSS and DRR either did not provide responses during the mapping exercise or relied on reporting from regional and local focal points who may have been overburdened or time-limited and thus unable to fully participate. As a result, the material in this review relevant to those agencies may not represent the entirety of their work. Third, this review was limited in its discussion of community resilience as a construct and as a set of DRR approaches. Because definitions and indicators for community resilience projects vary considerably, it was beyond the scope of this review to determine which projects or materials were relevant to MHPSS integration. Thus, many projects that may include relevant components in building community resilience were likely excluded. Fourth, the materials and projects that were considered in this review were limited to those available in English. Therefore, it is possible that other relevant materials or projects were not reviewed. Despite these limitations, the current review demonstrates a number of key themes in current practices of DRR and MHPSS integration and identifies areas for future development and focus in the field. 

## 5. Conclusions

The current review identified core themes of projects and resources relevant to the integration of MHPSS and DRR. These included themes of capacity building; preparedness; policy development, consensus building, and awareness raising; school- and child-focused initiatives; inclusive DRR; and resilience promotion. However, also identified through this review was a relative lack of guidance for integrating MHPSS and DRR and a limited consensus regarding what definitions, practices, and principles constitute this integration. 

Field experience suggests a significant need for integration of MHPSS and DRR practices in order to reduce risks of problematic mental health and psychosocial outcomes and to increase resilience to hazardous events. Such an integration may be beneficial to both fields in many ways. For instance, the MHPSS field may benefit from further mainstreaming given international support and buy-in for DRR and expansion of work to include prevention and preparedness while the DRR field may benefit from expanded approaches for promoting resilience and mental health and psychosocial well-being at multiple levels. The WHO has consistently advocated for the Building Back Better (BBB) approach, including with the publication of *Building Back Better: Sustainable Mental Health Care after Emergencies* [106]. The current undertaking emphasizes the value of expanding this concept of BBB to include the notion of ‘Building Better Before’ by including MHPSS before emergencies. If mental health and psychosocial well-being are incorporated within DRR efforts, both those who are affected and those who respond may be more likely to demonstrate resilience; engage actively in preparedness, response, and recovery efforts; and contribute to re-construction and re-establishment of societal functioning. Therefore, this review stands as a foundation for the forthcoming IASC MHPSS Reference Group’s *Mental Health and Psychosocial Components of Emergency and Disaster Risk Reduction: Framework for Action,* which aims to establish this needed consensus and guidance. This framework was developed to assist humanitarian aid, development, and Disaster Risk Management organizations and national and local governments and community actors in the delivery of a priority set of MHPSS-DRR actions. Included in the framework are sections linking MHPSS and DRR and outlining relevant activities, indicators, practical implementation tools, and case study examples. This document has undergone a multi-stage development process involving consultation with several experts in both the MHPSS and DRR fields and is due to be formally launched in 2020. Though only an initial step towards mainstreaming and promoting the integration of the MHPSS and DRR fields, this framework marks a significant attempt to address the gaps identified by the current review. 

## Figures and Tables

**Table 1:** Search strategy terms.

Terms:		Additional, Associated Text Words:	
1: Mental Health/Psychosocial/Psychological			
(a) Mental Health and Psychosocial Support		Preparedness	
(b) Psychological Health		Capacity building	
(c) Psychosocial Well-Being		Development	
(d) Psychological Resilience		Advocacy	
2: Disaster Risk Reduction			
(a) Disaster Risk Reduction (b) Hazard Reduction (c) Hazard Mitigation (d) Disaster Risk Management			
**Search Plan:**			
#1–1a and 2a #2–1a and 2b #3–1a and 2c #4–1a and 2d	#5–1b and 2a #6–1b and 2b #7–1b and 2c #8–1b and 2d	#9–1c and 2a #10–1c and 2b #11–1c and 2c #12–1c and 2d	#13–1d and 2a #14–1d and 2b #15–1d and 2c #16–1d and 2d
